# Governing complex societal problems: The impact of private on public regulation through technological change

**DOI:** 10.1111/rego.12314

**Published:** 2020-05-07

**Authors:** Nicolas Schmid, Leonore Haelg, Sebastian Sewerin, Tobias S. Schmidt, Irina Simmen

**Affiliations:** ^1^ Department of Humanities, Social and Political Sciences Energy Politics Group, ETH Zurich Zurich Switzerland

**Keywords:** energy efficiency, innovation, instrument interaction, policy change, policy design, policy feedback

## Abstract

When addressing complex societal problems, public regulation is increasingly complemented by private regulation. Extant literature has provided valuable insights into the effectiveness of such complex governance structures, with most empirical studies focusing on how public regulation influences private regulation. Conversely, the impact of private on public regulation is less well studied. Here, we investigate this impact with a focus on technological change as possible mechanism. Based on a case study of energy efficiency in buildings in Switzerland, we find evidence of a symbiotic interaction between public and private regulation that leads to ratcheting‐up of regulatory stringency. We identify technological change as the mechanism linking private and public regulation. We discuss the relevance of our findings for governance literature and regulators.

## 1 Introduction

Societies face increasingly complex and dynamic problems, with climate change being one of the most notable examples of such “super‐wicked” problems (Levin *et al*. 2012). To address these problems, complex governance structures have emerged in which traditional public regulations (i.e. mandatory command‐and‐control instruments) are complemented by private regulations (i.e. voluntary market‐based instruments) (Cashore 2002; Potoski & Prakash 2005; Loorbach 2010). This has resulted in complex and polycentric structures of governance (Levi‐Faur 2006; Meadowcroft 2007; Jordan *et al*. 2015). In such structures, regulatory governance occurs on various levels and involves both public and private actors who implement a wide range of public and private regulatory instruments (Hsu *et al*. 2019).^1^

Complementing public regulation with private regulation may increase the adaptability, accountability, and effectiveness of such governance structures (Keohane & Victor 2011) and potentially resolve the fundamental tension between regulatory rigidity and flexibility (Duit & Galaz 2008). At the same time, private regulation may erode the scope and authority of public regulation, resulting in less effective governance (Malhotra *et al*. 2019). Much of the existing governance literature is concerned with the question of whether these claims about the merits and pitfalls of private regulation hold true (Mattli & Büthe 2005; Bernstein & Cashore 2007; Green 2010; Schleifer 2017; van der Heijden 2020). Yet, since the effectiveness of private regulatory instruments also depends on their impact on other regulatory instruments, an emerging research stream focuses on the *interaction between public and private regulation* (Eberlein *et al*. 2014; Gulbrandsen 2014). This research has shown that public regulation plays a crucial role in facilitating the emergence, implementation, and enforcement of private regulation (Héritier & Eckert 2008). However, the reverse effect, that is the *impact of private regulation on public regulation*, remains understudied (Malhotra *et al*. 2019).

We argue that in order to fully comprehend the effectiveness of private regulation, an improved understanding of its impact on public regulation is required. To reach this understanding, the underlying mechanisms linking public and private regulation need to be fully understood (van der Heijden *et al*. 2019). While several mechanisms may exist, here we focus on the role of technological change and its feedback on regulation. Although technological change is a crucial factor in both creating and solving current societal challenges, its role for the interaction between regulatory instruments remains understudied.^2^ In extant regulation literature, technology is predominantly seen as an exogenous factor (Porter 2014; Snir & Ravid 2016) or a tool to improve the performance of regulatory instruments (Auld *et al*. 2010; Grabosky 2013; Fukuyama 2016) rather than a target of regulation itself. This conception of technological change as an exogenous factor arguably stems from the empirical focus of most studies on “disruptive” technologies (Hasselbalch 2018), such as information technologies (Yeung 2018; Kenney *et al*. 2019; Culpepper & Thelen 2020) and nanotechnologies (Sylvester *et al*. 2009). For such technologies, regulation may be more *reactive* – as opposed to actively steering innovation – and less influential than in the case of “incremental” technological change, such as in the building sector. In contrast to this conception, and in line with innovation studies (Hoppmann *et al*. 2014; Kivimaa & Kern 2016; Schmidt & Sewerin 2018), we conceptualize technology as the *target outcome* of a policy, meaning that the goal of the policy is to induce technological change. We draw on policy feedback literature (e.g. Pierson 2000; Béland & Schlager 2019; Schmid *et al*. 2019) to discuss how private regulation can, over time, feed back into public regulation through technological change. In this context, technological change may constitute one of several important *mechanisms* that explains the impact of private regulation on public regulation. We aim to complement existing governance literature by exploring the role of technological change as a mechanism and by asking the following research question: *How does private regulation influence public regulation, and how does technological change affect this relationship*?

To address this question, we build on theoretical discussions in governance literature regarding regulatory instrument interaction as well as innovation studies and policy feedback theory. Empirically, we focus on the case of energy efficiency in the Swiss building sector, which provides a suitable case for an exploratory theory‐building study. To understand the impact of private regulation on public regulation, we employ a mixed‐methods approach to analyze changes in the *regulatory stringency* of two Swiss regulatory instruments over time – namely, public building standards and a private building label.^3^ First, we construct a novel and extensive dataset on both public regulation across 23 Swiss Cantons (i.e. subnational jurisdictions) and private regulation over more than 30 and 20 years, respectively. Second, we conducted 27 semistructured expert interviews to understand the drivers behind public and private regulatory stringency. We find that the stringency of both public and private regulatory instruments increases over time, with the latter being more stringent. Crucially, our analysis provides substantial evidence for a symbiotic interaction between public and private regulatory instruments. Private regulation can, through fostering technological innovation in niches, drive higher stringency in public regulation at a subsequent time. Thus, we show that, under certain conditions, combining public and private regulation can increase overall governance performance through a mutual ratcheting‐up process. This finding puts into context the ongoing debates in regulatory and governance studies about whether public or private regulation is more effective in isolation. Our main finding also highlights the role of technological change as a mechanism explaining this symbiotic ratcheting‐up process: Technological change induced by private regulation increased the political feasibility of more stringent public regulation by expanding the availability of technically feasible and economically affordable technologies, which constituted positive feedback effects in the regulatory process. Further, we present contextual factors that moderate instrument interaction through technological change – namely, institutional and political differences between cantons and technological differences between building components. Finally, we discuss the theoretical relevance of our findings for governance literature as well as the practical relevance of our findings for regulators.

## 2 Instrument interaction and the role of technological change therein

In light of increasingly complex societal problems, various research streams have investigated the shift “from government to governance”, that is from sole state‐authority in governing to the involvement of nonstate stakeholders (Cashore 2002; Potoski & Prakash 2005; Levi‐Faur 2012; Steurer 2013; Eberlein *et al*. 2014; Fukuyama 2016). Generally, most studies investigate the nature and effectiveness of private regulatory instruments (Szulecki *et al*. 2011; Auld *et al*. 2015; Darnall *et al*. 2017; Dietz *et al*. 2019; van der Heijden 2020). The debate regarding whether private regulatory instruments have a positive, neutral, or negative effect on governance performance is still ongoing (Howlett & Rayner 2007; Carrigan & Coglianese 2011; Hoffmann 2011; Matschoss & Repo 2018; Chan *et al*. 2019). The extant literature is mostly comparing private regulatory instruments across jurisdictions or sectors (Judge‐Lord *et al*. 2020; van der Heijden 2020). However, these studies have resulted in inconclusive empirical findings (Hsu *et al*. 2019). Reaching more conclusive findings might necessitate research designs that take into account the *interaction* between public and private regulatory instruments.

## 3 Regulatory instrument interaction in governance literature

A growing research stream therefore specifically focuses on these interactions (Gulbrandsen 2014; Andanova *et al*. 2017; Bartley 2018; Dietz *et al*. 2019; Trencher & van der Heijden 2019), showing that the emergence, implementation, and enforcement of private regulation is strongly influenced by existing public regulatory instruments (Héritier & Lehmkuhl 2008; Vogel 2008; Verbruggen 2013; Auld *et al*. 2014; van der Heijden 2015). Although this research has provided important insights into instrument interaction, existing studies primarily focus on “one‐way interactions” from public to private regulation (Trencher & van der Heijden 2019). We argue that in order to fully understand the nature and effectiveness of private regulation, more empirical research into the reverse effect – that is private on public regulation – is needed (Arcuri 2015; Malhotra *et al*. 2019). On a theoretical level, public regulation may benefit from engaging with private regulation since it enables regulators to combine the competencies of public and private actors (Abrams *et al*. 2018). While some scholars suggest that private regulation enables experimentation and learning (Matschoss & Repo 2018), other scholars argue that private regulation may “crowd out” or preempt more stringent public regulation (Malhotra *et al*. 2019) or even undermine public regulation altogether (Baron 2014). This could potentially limit the overall scope of regulation, that is, how much of the relevant regulatory targets are addressed. Such crowding out may be even more prevalent with the growing complexity of societal problems: The regulation of “super‐wicked” problems (Levin *et al*. 2012) may be biased toward the preferences of private actors because of information asymmetry and principal–agent problems between public administrations and private actors (Héritier & Eckert 2008; McCarty 2015). These asymmetries may be even more pronounced if technology is involved in addressing the societal problem (Mattli & Büthe 2005; Eberlein 2008), such as in the case of climate change mitigation through technological change (Gilligan & Vandenbergh 2020). Hence, the effect of private regulation on public regulation depends also on the governance capacity of public regulators (Knill & Lehmkuhl 2002; Howlett & Rayner 2006).

### 3.1 Technological change as mechanism linking public and private regulatory instruments

**Figure 1:**
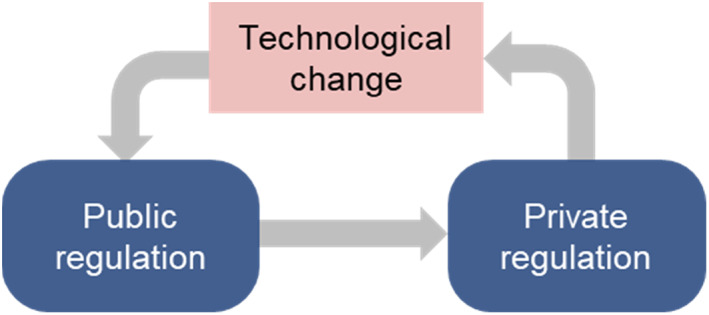
The impact of private regulation on public regulation through the mechanism of technological change.

Given the important role of technology in both creating and solving problems in various regulatory fields (Jaffe *et al*. 2002), it is surprising that the influence of technological change in shaping the impact of private regulation on public regulation remains largely uncovered (Auld *et al*. 2010). Although the role of private regulation for the regulation of technology has been central to academic debates in the 1970s and 1980s (Stigler 1971; Buchanan & Tullock 1975; Quirk 1982; Bailey 1987), it has been absent from more recent governance literature: If considered at all in this literature, technology is mostly seen as an exogenous factor that remains relatively independent from regulation (Porter 2014; Snir & Ravid 2016; Schmid *et al*. 2019). This conception of the role of technology arguably stems from the empirical focus of most studies on “disruptive” technologies (Hasselbalch 2018), such as information technologies (Yeung 2018; Kenney *et al*. 2019; Culpepper & Thelen 2020) and nanotechnologies (Sylvester *et al*. 2009). For such technologies, regulation may be more *reactive* – as opposed to actively steering innovation – and less influential than in the case of “incremental” technological change, such as in the building sector. Other scholars understand technology as a tool, that is *mechanic*, used to improve the performance of regulatory instruments (Grabosky 2013; Fukuyama 2016). For instance, Auld *et al*. (2010) investigate how innovative GPS tracking technology or DNA testing can improve the environmental performance of private regulation in complex global supply chains. The authors argue that private regulation “may play a role as technology incubator, potentially facilitating and fostering, rather than bypassing, traditional public policy efforts at the domestic or global levels” (p. 24). However, they acknowledge that how exactly private regulation influences public regulation through technology remains an open question for future research. Here, we attempt to address this question by conceptualizing technology as a *target outcome* rather than a tool or mechanic of regulation. In this view, which is in line with innovation studies literature (e.g. Hoppmann *et al*. 2014; Kivimaa & Kern 2016; Schmidt & Sewerin 2018), technological change can be seen as a mechanism linking different regulatory instruments over time. Insights from policy feedback literature can help to further describe how exactly technology may connect public to private regulation. The classical argument of this literature is that changes in regulation at *t*_*i*_ can affect the politics of subsequent changes in regulation at *t*_*i* + 1_ (Pierson 2000; Jordan & Matt 2014). Recent studies have looked at the role of technological change in such feedback processes. Based on a case study of the German electricity sector, Schmid *et al*. (2019) have developed a framework in which technological change is conceptualized as a regulatory outcome that influences subsequent political processes through feedback mechanisms. Meckling *et al*. (2015) have argued that industrial regulation can create positive feedback through the creation of green industries that support subsequent regulation targeted at the decarbonization of the economy. Importantly, this literature suggests that such positive feedback can be intentionally created by smart regulatory design. Regulators that aim at increasing the political feasibility and stickiness of subsequent regulatory instruments in a regulatory mix (Jordan & Matt 2014; Howlett 2019) can, for example, follow an instrument sequencing approach (Taeihagh *et al*. 2013; Meckling *et al*. 2015, 2017; Schmidt *et al*. 2019). These insights from literature on technological innovation and policy feedback have important implications for theories of regulatory governance: In many regulatory fields, both public and private regulation influence technology, meaning that technological change may strongly mediate instrument interaction and should thus be conceptualized as a mechanism rather than tool or mechanic (see Fig. 1 below).

Hence, explicitly conceptualizing technological change as a mechanism may help analyze the impact of private regulation on public regulation. Considering this impact may enable more valid assessments of overall governance effectiveness. In a theory‐building endeavor, this paper explores whether and how private regulation has an impact on public regulation through the mechanism of technological change. Note that we focus on changes in regulatory stringency (Judge‐Lord *et al*. 2020) in order to capture instrument interaction.

## 4 Case selection and methodology

### 4.1 Case selection

To analyze the effects of private on public regulation through technological change, we chose the field of *energy efficiency of buildings*, for three reasons. First, our case is suitable for investigating the link between technology and regulation as increasing efficiency in the building sector represents a very complex societal problem for which technological change represents the most important solution (International Panel on Climate Change [IPCC] 2014).^4^ Yet, multiple barriers hamper the diffusion of energy efficient technologies. Such barriers include the high upfront cost of technologies, high transaction costs, strong lock‐in effects, and the principal–tenant problem (Gillingham & Palmery 2014; Rosenow *et al*. 2017). Because of these barriers, public and private regulations are needed to increase energy efficiency in the building sector (Noailly 2012; Asensio & Delmas 2017; Girod *et al*. 2017). Note that most current regulatory instruments target new buildings only. As a consequence, the focus of our paper is on the governance of new buildings and we thus disregard regulations on building retrofits. Second, from an instrument interaction perspective, the case of energy efficiency remains a poorly studied phenomenon as most studies on energy efficiency focus on the effectiveness of regulatory instruments (but see Kern *et al*. 2017; Rosenow *et al*. 2017; Trencher & van der Heijden 2019). Third, increasing the energy efficiency of buildings can substantially contribute to climate change mitigation. The building sector accounts for 19 percent of direct and indirect global greenhouse gas emissions, and it is the regulatory field with the highest potential for energy efficiency improvements (International Panel on Climate Change [IPCC] 2014). Given the length of investment cycles in this sector, fast regulatory intervention is important for climate change mitigation.

Further, we chose the case of *Switzerland* for three reasons. First, energy efficiency governance in Switzerland is effective compared to other countries, as the final energy consumption of buildings has decreased for new buildings (Kemmler *et al*. 2018). Second, the empirical case offers a high level of data variance regarding public and private regulatory instruments due to substantial and long‐lasting regulatory activity in this field. This facilitates the analysis of instrument interaction over time. Public building standards were established early on (i.e. starting after the oil crises in the 1970s) and subsequently diffused across jurisdictions (Strebel 2011; Strebel & Widmer 2012; Sager *et al*. 2014). Twenty‐six different public building standards are enacted in Switzerland because subnational entities are responsible for building regulation. The federal level intervenes only as a facilitator of coordination between cantons (Casado‐Asensio & Steurer 2016). Although efforts to align standards through model regulations were expanded over time, these model regulations offer extensive freedom in relation to the speed and extent of their adoption (Strebel 2011; Grösser 2012). Hence, standards are heterogeneous across jurisdictions and time. In addition, standards contain different requirements depending on the target technology (e.g. windows or insulation materials for walls). Besides public regulation, the existence of an influential private regulatory instrument makes it possible to analyze the impact of private regulation on public regulation. An association called Minergie was founded in 1998 by private individuals, together with firms and banks, and received the support of cantonal building administrations (Aeberhard 2018; Lange *et al*. 2019). Since its creation, Minergie has issued a nationwide label to certify buildings – independent from other international labels, such as the Leadership in Energy and Environmental Design label. The purpose of this private building label was to build a trusted trademark with enforced compliance in order to create incentives for the diffusion of highly energy‐efficient technologies. Third, the existence of a strong and diversified Swiss construction industry means that technological change happens mostly within the jurisdictional borders (Bättig & Ziegler 2009). Although we cannot exclude spillover effects from other countries, the leadership of local firms in energy‐efficiency technologies on the Swiss market means that such effects should be marginal.

### 4.2 Methods and data

In this study, we applied a mixed‐methods approach combining quantitative analyses on public and private regulatory instruments with qualitative analysis from semistructured interviews. We proceeded in two steps. First, in a major effort of data collection, we constructed a novel cross‐sectional and longitudinal dataset on the stringency of both public and private regulatory instruments. Public building standards set requirements on the minimum level of insulation of different building components, measured with the so‐called *U* value. The *U* value represents the amount of heat that is transferred by the surface of a building component at a given temperature difference (measured in W/m^2^K). Accordingly, the *U* value decreases with increasing regulatory stringency. The data were collected from cantonal energy departments, construction departments, and energy offices. In total, we obtained data from 23 of the 26 cantons, for the period between 1975 and 2018. For the private building label, we collected the stringency values reported as systemic efficiency values as well as, where applicable, the *U* values for individual building components. In the second step, we conducted semistructured interviews with experts and stakeholders to understand the drivers behind changes in public and private regulatory instrument stringency. The interviewees listed in Table 1 were identified by desk research following a combination of positional, decisional, and reputational approaches (Knoke 1996). We conducted 20 in‐depth expert interviews, with each interview lasting between 40 and 100 minutes. Additionally, we conducted seven interviews, with each interview lasting between 10 and 30 minutes, at a construction industry fair in Lucerne. All interviews took place between August and December 2018.

**Table 1:** List of interviewees

Person	Category	Description
1	Civil servant	Director of the Department of Energy and Environment, the canton of Bern
2	Civil servant	Employee at the cantonal energy bureau, the canton of Zurich
3	Civil servant	Former employee at the cantonal energy bureau, the canton of Grisons
4	Civil servant	Employee of the Swiss Federal Office of Energy
5	Civil servant	Technical expert at the Intercantonal Conference of Energy Directors (ICED)
6	Civil servant	Director of the Department of Energy and Environment, the canton of Neuchatel
		Director of the Conference of the Swiss–French Energy Departments (CRDE)
7	Civil servant	Director of the Department of Energy and Environment, the canton of Basel‐City
8	Policy consultant	Consultant on energy efficiency regulation in Switzerland
9	Politician	Head of the cantonal energy bureau, the canton of Grisons
		President of ICED (2003–2010)
10	Representative of individual company	Employee of a Swiss wood heating company
11	Representative of individual company	CEO of a Swiss heat pump company
12	Representative of individual company	CEO of a Swiss building and assembly company
13	Representative of individual company	Employee of a Swiss house ventilation company
14	Representative of individual company	Employee of a Swiss insulation material company
15	Representative of individual company	Employee of a Swiss window manufacturing company
16	Representative of individual company	Employee of a Swiss house ventilation company
17	Representative of industry association	Head of the Swiss Association for Windows and Facades (SZFF)
18	Representative of industry association	Member of the Swiss Association of Engineering and Architecture (SIA)
19	Representative of industry association	Member of the Swiss Association of Engineering and Architecture (SIA)
20	Representative of industry association	Employee of the Swiss Association of Building Technology (suissetec)
21	Representative of industry association	Employee of the Swiss Association of Home Owners (HEV)
22	Representative of industry association	Employee of the Swiss Association of Heat Pumps (FWS)
23	Representative of industry association	Employee of the Swiss Association of Building Envelopes
24	Representative of private building label	Co‐founder of Minergie
25	Representative of private building label	Former director of Minergie
26	Representative of private building label	Director of Minergie
27	Representative of private building label	Former technical advisor to Minergie

## 5 Results

### 5.1 The Swiss building sector: Dynamics in public and private regulatory stringency

**Figure 2:**
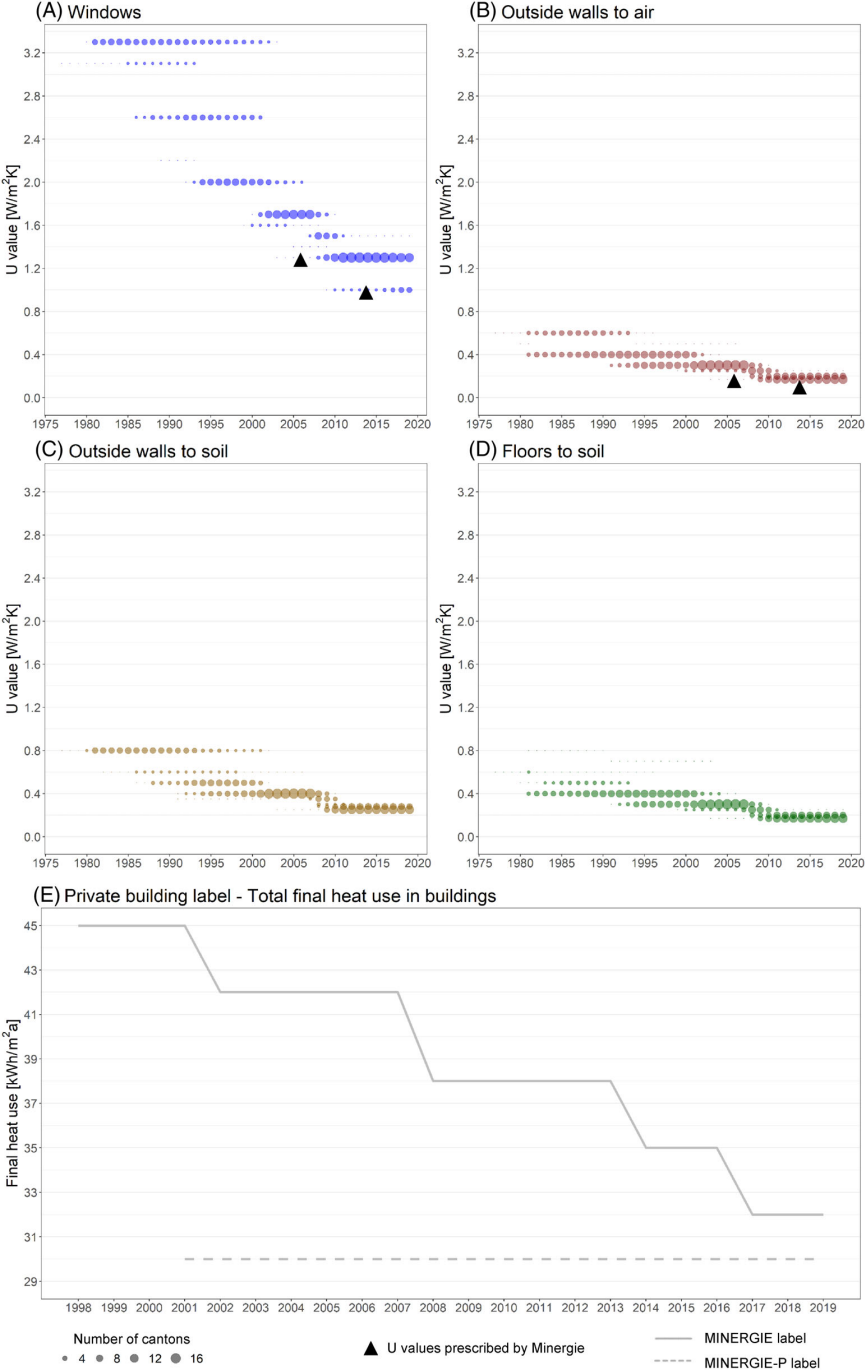
Public building standards for (a) windows (blue); (b) outside walls to air (red); (c) outside walls to soil (ocher); and (d) floors to soil (green) for each year. Decreasing values correspond to increasing stringency of the standard and label. The bubble size corresponds to the number of cantons with the same value (*n*_total_ = 23). The black triangles correspond to the *U* values prescribed by the private building label. (e) Private building label's prescribed total final heat use in new residential buildings for standard buildings (solid line) and for buildings meeting specific requirements regarding the building shell (dashed line). The final heat use includes the weighted energy use for heating, ventilation, air conditioning, and hot water.

Figure 2 summarizes the results of our data collection and shows the evolution of public building standards for different building components (Fig. 2a–d), as well as the evolution of the stringency of the private building label (Fig. 2e). Based on this novel dataset, three general observations can be made. First, public building standards have become more stringent and more aligned across cantons over time. The standards were adapted multiple times over the past 40 years in order to increase their stringency. Especially for windows, steep reductions in *U* values and thus increases in stringency can be detected (i.e. from 3.0 W/m^2^K in 1977 to 1.0 W/m^2^K in 2018; see Fig. 2a). Although weaker, increases in stringency can also be detected for walls and floors (Fig. 2b–d). Additionally, public standards have become more aligned over time. Early on, many different *U* values existed for each technology within Switzerland, but their number as well as their range decreased later (e.g. windows had five different prescribed *U* values between 2.0 and 3.3 W/m^2^K in 1992 and three between 1.0 and 1.5 W/m^2^K in 2011; see Fig. 2a).^5^ Second, we find large differences in public building standards across target technologies in terms of the stringency of standards and changes in stringency over time. On the one hand, standards for windows were much higher at the outset than for walls and floors (e.g. *U* values between 3.1 and 3.3 W/m^2^K for windows and *U* values between 0.4 and 0.8 W/m^2^K for walls and floors in the 1980s; see Fig. 2a–c). On the other hand, the standards for windows were adapted more often and decreased at a higher rate than the standards for walls and floors (e.g. 11 different *U* values for windows and seven different *U* values for outside walls to air over the complete period; see Fig. 2a,b). In total, the standards for windows decreased to a greater extent (i.e. from 3.0 W/m^2^K in 1977 to 1.0 W/m^2^K in 2018) compared to a smaller decrease in walls to air (i.e. from 0.6 W/m^2^K in 1977 to 0.2 W/m^2^K in 2018). Third, as visualized in Figure 2e, the private Minergie label has become more stringent over time, similar to public building standards (i.e. from 42 kWh/m^2^a in 1998 to 35 kWh/m^2^a in 2019).^6^ Depicted as a triangle in Figure 2a,b, the Minergie *U* values are lower in comparison to public building standards, and they predate the subsequent increase in the stringency of standards. In 2001, a more stringent additional label, Minergie‐P, was created.

### 5.2 Changes in public and private regulation and the role of technological change

**Figure 3:**
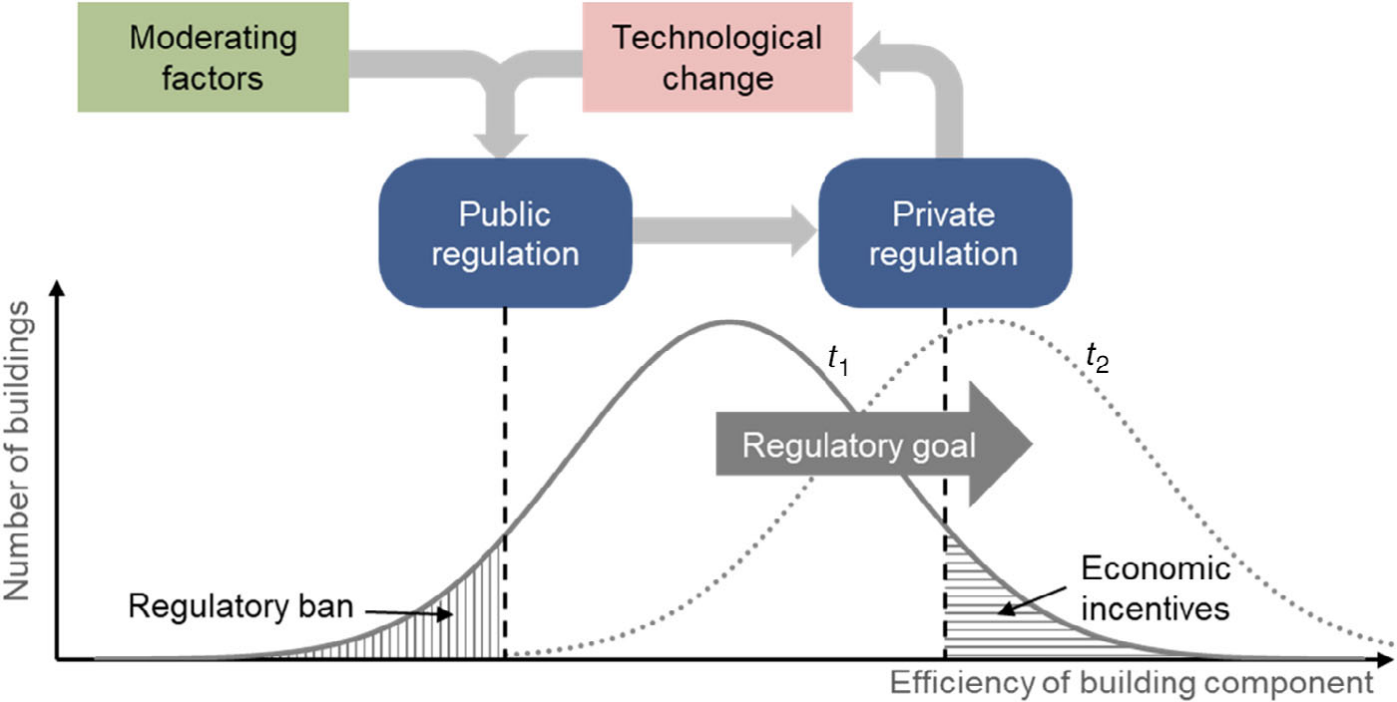
Interaction between public and private regulation through the mechanism of technological change. The regulatory goal to increase overall efficiency of building components over time (time 1 to time 2) is driven, on the one hand, by the private building label, which increases the economic incentives and feasibility of more efficient building components, and on the other hand, by public building standards, which ban inefficient building components (adapted from Interviewee 2).

The regulatory goal of both public and private regulatory instruments is to phase out less‐efficient and phase in better‐performing building components, thereby shifting the overall distribution of building components toward higher efficiency (see red arrow in Fig. 3). To do so, the stringency of these instruments has to increase over time. In the following section, we discuss whether and how technological change has acted as a mechanism in explaining the increase in stringency of these instruments as well as their interaction. Figure 3 summarizes the three arguments below.

First, technological change was important in shaping public building standards. These standards set mandatory efficiency levels for components of new buildings thus effectively banning the most inefficient building components (see Fig. 3). In setting these standards, regulators could only adopt stringency levels for which corresponding building parts were technically feasible and economically available (Interviewees 1, 6). Interviewee 2 stated in this regard that “only when a technological development is acknowledged by the architects and engineers, you can start drafting a new standard. Politicians will ask these experts' opinion and, only when these experts know the new technology, you will obtain a majority.” In the early phase of building regulation, efficiency gains were achieved by incrementally improving existing technologies. For instance, in the 1970s and early 1980s, two‐glazed windows with *U* values of around 2.5 W/m^2^K were mainly used for new buildings. At the same time, cantons with standards mandated values of between 3.1 and 3.3 W/m^2^K. In the following years, improvements in glass and filling gas led to reductions in the thermal conductivity of windows (Interviewee 2), and standards were consequentially lowered to values of around 2.0 W/m^2^K. Similar trends can be reported for wall insulation: Early efficiency gains for walls were achieved by increasing the thickness of the insulation material (Interviewees 2, 5). However, neither the efficiency of two‐glazed windows nor wall thickness could be indefinitely increased for technical reasons and due to opposition by artisans and architects (Interviewees 1, 2, 15). In the 1990s, however, innovative firms pushed through technological change. On the one hand, they introduced three‐glazed windows that substantially reduced the *U* values (Interviewee 1). On the other hand, manufacturers of insulation material adapted their materials and could thus provide some efficiency gains per volume of insulation material (Interviewees 2, 14). Once these technologies were widely diffused on the market, regulators could increase the regulatory stringency of public building standards to account for this development. As stated by Interviewee 2, “the standards represent the state of the technology. […] In some years, they will have developed further, and we will need to revise the standards to account for the [new] state of the technology.”

Second, technological change itself was shaped and triggered by the private building label. As argued above, the diffusion of new technologies led to increasingly stringent public building standards. However, technological change did not occur in a vacuum. Rather, the most important driver of innovation in and diffusion of energy efficient technologies in the Swiss building sector was the private building label. The label was introduced in 1998 with the specific intention to foster innovation in the construction industry and diffuse new energy‐efficient technologies in the market (Aeberhard 2018) by creating economic incentives for and improving the economic feasibility of more efficient building components (see Fig. 3). From the beginning, the private building label received strong support from administrative cantonal actors, who perceived the label as a complementary technology‐pull instrument to the demand‐push public building standards (Interviewee 6). The label's effects on technological change were manifold. It created a high‐performing benchmark and premium for the industry thus ensuring a safe market for the industry's high‐tech products (Interviewees 12, 13, 8). Interviewee 24 stated, “Minergie created a market where there was no market before.” By continuously increasing the label's stringency, the producers of energy efficient technologies were additionally incentivized to keep improving their technologies. By educating and certifying artisans and architects, the private building label not only ensured the competent installation and use of the new technologies but also offered these actors more network and reputation benefits compared to artisans and architects relying on conventional technologies (Interviewees 1, 24). Hence, it was an additional source of value creation and quality control for several actors along the supply chain in the building sector. Interviewee 2 stated, “Minergie managed to create added value. A Minergie‐certified house can be sold at a higher price per square meter than a house without the label.” The private building label also decreased the information asymmetry between artisans and homeowners (Interviewee 27). Overall, the private building label incentivized firms to innovate and homeowners to invest in new high‐end building components, such as three‐glazed windows and thicker wall insulation. Only thanks to this niche market could new and efficient technologies diffuse widely and drive down their learning curves.

Third, and bringing both findings together, technological change can explain the interaction between public and private regulatory instruments (see Fig. 3). The private building label influenced public building standards by showing what was technically feasible and improving the economic viability of high‐end products (Interviewees 2, 21). Technological change triggered by this niche market could, in turn, result in more stringent public building standards. Interviewee 5 stated in this regard, “Minergie was a guiding player that paved the way for cantonal energy legislation. They brought products and technologies into large application that were back then not standard yet. […] This fueled technological change and led to a technological standard that could be enacted in our Energy Law with a time lag.” Interestingly, public regulation, in turn, influenced the private building label. As a result of increasing public regulatory stringency, the association issuing the label needed to increase its stringency in order to maintain its benchmark function (Interviewees 4, 25). Interviewee 4 stated in this regard, “around 2008, [the canton of] Basel‐Stadt tightened the mandatory standard for the building envelope by 10% which meant that the requirements by the private label had to become more stringent as well because it cannot be less strict than cantonal legislation. Thus, the two policies amplified each other.” Further, Interviewee 4 summarized this interaction between public and private regulation as follows, “the individual policies determine each other. With its goal to try out new things, the private label sets the benchmark for the standards. The public standard always comes last. They prescribe the use of state‐of‐the‐art technology and thus enforce them on the last 20–30% of constructors.” Official documents issued by cantonal and intercantonal institutions (EnDK 2008, 2015; BEG 2009; AWEL 2018) confirm these findings from interviews: For instance, the private label Minergie is consistently mentioned as benchmark‐setter for public regulation.

### 5.3 Sub‐national variations and differences across technologies: The moderating factors

In addition to the previously presented symbiotic relationship between private regulation, technological change, and public regulation, we found moderating factors that influenced this interaction (see Fig. 3). These factors are related to the complexity and contingency inherent in the regulatory field of energy efficiency in Switzerland. The public building standards differ across subnational entities in terms of their stringency, the year of implementation, and the number of adaptations (see Fig. 2a–d). These differences can be explained by political and institutional factors as well as the influence of additional regulatory instruments (Interviewees 4, 25). First, in the early phase of standard implementation, frontrunner cantons often exhibited enhanced institutional capacities within their cantonal administrations (Interviewees 2, 4, 5, 9), which were capable of elaborating on and implementing such legislation. Whether a canton was a frontrunner in standard implementation also depended on political conditions, such as the party affiliation of the responsible minister or the composition of the cantonal parliament as well as the locus of implementation (i.e. whether it was enacted within the law or in subordinate ordinances) (Interviewees 4, 9). The increasing alignment between public building standards in the latter years is a result of regulatory harmonization and diffusion efforts by intercantonal institutions, notably the Intercantonal Conference of Energy Directors (Strebel 2011). Second, the energy bureaus of several cantons as well as the Federal Office of Energy implemented regulatory instruments other than public standards and the private label. Less influential than the private building label, these additional instruments were introduced mostly at the cantonal level. They included pilot and demonstration programs (Interviewees 4, 5, 6), subsidies for novel energy‐efficient technologies (Interviewees 2, 4, 5, 6), financial incentives for homeowners to retrofit specific building parts (Interviewee 6), and information and education campaigns for artisans (Interviewee 3). Overall, the differences regarding political and institutional factors as well as additional regulatory instruments can explain the divergence in stringency, implementation, and adaptations across cantons.

Besides the intercantonal differences, Figure 2 also highlights the differences in regulatory stringency, implementation, and adaptations across technologies. These differences (e.g. between windows and wall insulation) can be explained by the different starting points and learning potentials of individual technologies as well as by the industry structure underlying these technologies. First, technology differences account for variation in regulatory stringency across technologies. As Interviewee 2 stated, “[large] modifications [of the *U* values for windows] were enabled by technological progress. We did not treat windows more strictly, but we had more possibilities.” As Interviewee 5 stated, “the technological leaps did not take place at the same frequency or to the same decisive extent for insulation material than for windows.” With the introduction of three‐glazed windows in the 1990s, the *U* value of windows could be considerably reduced (Interviewee 2). Similar improvements were impossible for wall and floor insulation (Interviewee 2). Hence, in the case of wall insulation, the technical limits compromised further ratcheting up of regulatory stringency (Interviewee 14). Second, another factor for these technology differences is the structure of the Swiss construction industry. For instance, the value chain of windows includes a wide range of actors with different interest: On the one hand, Swiss glass manufacturers were highly interested in more stringent *U* values, as the latter resulted in the introduction of three‐glazed windows and thus increased glass sales (Interviewees 2, 5, 25). On the other hand, local carpenters producing window frames opposed this technological change, since they had to invest in new equipment and expertise (Interviewees 2, 4, 5, 17, 25). They effectively resisted *U* value adaptations for windows in smaller cantons (Interviewees 2, 4). Conversely, Swiss companies leading in insulation material production were interested in reductions of the *U* value (Interviewees 2, 5) and were even active within Minergie (Interviewees 4, 25). Yet, some artisans mounting the insulation material as well as architects all opposed *U* value reductions, since thicker walls meant changes in their procedures and less freedom in the design, respectively (Interviewees 2, 3, 4, 18). Hence, the ratcheting‐up process between the public and private regulatory instruments was conditioned both by institutional and political factors and technology‐specific characteristics of the targeted building components.

## 6 Discussion and conclusion

We investigated the long‐term interaction between regulatory instruments, specifically focusing on the impact of private on public regulation. We explored whether technological change can explain this impact. Based on a case study on energy efficiency in buildings in Switzerland, we find that both public and private regulatory instruments are becoming increasingly stringent with the same directionality. Over time, the public regulatory instruments' stringency approaches the private instrument's stringency. Hence, our results indicate an *increasing convergence* between regulatory instruments (Judge‐Lord *et al*. 2020). Further, the interaction between public and private regulation suggests a symbiotic relationship. While the public instrument ensures a broad *scope* of the governance structure, the private instrument set the *benchmark* for the direction of changes in instrument stringency. Our findings indicate that the *mechanism* behind this relationship is positive feedback from technological change: The private regulatory instrument induced technological change in the building sector by creating niche markets for new high‐end technologies. These niche markets enabled innovation in and diffusion of new energy‐efficient technologies, such as three‐glazed windows and improved wall insulation. These technologies became cheaper due to increased market diffusion and resulted in economies of scale and learning effects for the technology manufacturers, installers, and users (Dosi 1982; Sandén & Azar 2005). This wider range of technically feasible options and economically affordable technologies, in turn, constituted positive feedback effects for subsequent regulatory change.

On a more abstract level, our analysis reveals a successful case of a *ratcheting‐up process* between both public and private regulation (Cashore *et al*. 2007; Overdevest 2010; Meckling *et al*. 2017; Pahle *et al*. 2018; Judge‐Lord *et al*. 2020). As a consequence of technological change induced by private regulation, regulators were able to subsequently increase the stringency of public regulation. Hence, conversely to skepticism toward the benefits of private regulation in governance literature (Mayer & Gereffi 2010), we show that private regulatory instruments can foster public regulation. However, this successful example of ratcheting‐up depends on the capacity of private regulation to actually trigger technological change. In the absence of such an effect, we would not expect the necessary positive feedback to enable a ratcheting‐up between public and private regulation. These new empirical and theoretical insights were possible by focusing on regulation‐induced technological change. The case of energy efficiency technologies in the building sector is therefore distinct from studying “disruptive” technologies (Hasselbalch 2018; Yeung 2018; Kenney *et al*. 2019) for which regulation plays a more reactive than active role.

Importantly, as our results also highlight, instrument interaction through technological change does not happen in a vacuum, but it is moderated by political and institutional factors as well as differences between technologies. First, whether a symbiotic interaction between public and private instruments exists depends on the *governance capacity* of public and private regulatory instruments, that is “the formal and factual capability of public or private actors to define the content of public goods and to shape the social, economic, and political processes by which these goods are provided” (Knill & Lehmkuhl 2002, p. 43). In our case, both public and private regulatory instruments have high governance capacity, which makes it an example of *regulated self‐regulation* (Knill & Lehmkuhl 2002). As such, it combines the rigidity of public regulation with the flexibility of private regulation, resulting in a better adaptive capacity for complex problems. According to Duit and Galaz (2008), the adaptive capacity of governance is understood as a combination of exploitation (i.e. the capacity to benefit from existing forms of collective action) and exploration (i.e. the capacity of governance to nurture learning and experimentation). Second, the specific type of governance capacities and interactions depend on three contextual factors (Knill & Lehmkuhl 2002): the *congruence* between the problem scope (local, national, or global) and the governance structure (local, national, or international regulatory bodies); the *type of problem* at hand (degree of complexity and coordination/conflict patterns); and finally, the *institutional context* (rules and norms influencing actor behavior and decision processes). Although technological change is generally a global phenomenon, technological change in the building sector remains highly localized in terms of supply chains, actor constellations, and specific regional and national use of environments and regulation. Hence, national and regional regulatory instruments are arguably congruent to technological change in such a localized industry. Concerning the type of problem at hand, technological change represents a complex governance challenge, especially in the construction industry, which is characterized by relatively complex supply chains, a high diversity and multitude of actors involved, and technologies adapted to the specific use environment. As commonly argued, in regulatory fields with such high complexity, the integration of private regulation is likely to complement public regulation by adding flexibility to the rigidity of public regulation (Duit & Galaz 2008). Finally, the institutional setting in Switzerland is characterized by consensus‐based regulation, dense regulatory networks (Sager *et al*. 2014), and the inclusion of interest groups in the regulatory process (Maggetti 2014), which is conducive to regulated self‐regulation (Maggetti *et al*. 2011). In the case of energy efficiency governance in Switzerland, both the governance capacity and the three contextual factors discussed above enabled the symbiotic interaction between public and private regulation through technological change. We encourage future research that replicates our approach of studying the influence of private on public regulatory instruments in other regulatory fields and across jurisdictions. Such research could also provide more evidence on the necessary pre‐conditions for a symbiotic instrument interaction through technological change.

Finally, our paper contains an important message for regulators in fields where technology is relevant: Depending on governance capacity and contextual factors, private regulation can serve as a lever to increase regulatory stringency over time through the mechanism of technological change. As such, the intentional introduction of private regulatory instruments could increase the political feasibility of public regulation in fields that are characterized by inertia and lock‐in (Cashore *et al*. 2007; Levin *et al*. 2012; Meckling *et al*. 2015, 2017; Judge‐Lord *et al*. 2020). Successfully leveraging this rationale, however, requires *technology‐smart* governance (Beuse *et al*. 2018): Depending on the characteristics and maturity of a technology, as well as the capabilities of the local industry, different combinations of public and private regulatory instruments might be more effective and politically feasible than others (Schmidt & Huenteler 2016; Breetz *et al*. 2018). Our findings suggest that such technology smartness necessitates a robust governance structure combining regulatory rigidness and flexibility (Duit & Galaz 2008).^7^ We argue that such a differentiated approach to the role and the respective merits of public and private regulation is needed to better understand governance structures for solving complex societal problems such as climate change.

Endnotes.

## Endnotes

^1^
In our paper, we use governance and regulatory governance interchangeably, following Levi‐Faur's (2012) definition of regulatory governance as the organized attempt to steer the behavior of targeted actors through both public and private regulatory instruments (see also Eberlein *et al*. 2014).

^2^
Here, technological change is understood as the invention, innovation, and large‐scale diffusion of new technologies (Jaffe *et al*. 2002).

^3^
We define regulatory stringency along the lines of Carley and Miller (2012) as different levels of standards or limit values in regulatory instruments.

^4^
In this paper, we use Pérez‐Lombard *et al*.'s (2013) definition for energy efficiency, who state that “efficiency is the ability to achieve a desired result wasting minimum resources.” Energy efficiency is thus always a relative number as opposed to energy savings, which indicate the absolute reduction in energy use.

^5^
Note that the almost linear reduction of *U* values observed in Figure 2 represents above business‐as‐usual technological progress, which tends to slow down over time (Nagy *et al*. 2013), including in building technologies (Jakob & Madlener 2004).

^6^
Due to data availability, we do not report specific *U* values for the private building label but show the more systemic measure of total final heat use per square meter per year (kWh/m^2^a). *U* values mandated by the private building label for specific building components are only available for some years and technologies.

^7^
Findings of this paper should also be of interest for the policy design literature, which is concerned with how regulatory instruments can be designed to ensure their political feasibility and durability (Jordan & Matt 2014; Capano & Woo 2017; Haelg *et al*. 2019; Howlett 2019).
